# Using data from a private provider of telemedicine to assess the severity of the early 2021 Covid-19 wave in Brazil

**DOI:** 10.1590/1414-431X2022e11959

**Published:** 2022-06-22

**Authors:** P.M. Barbosa, F.C. da Silva, G.M.C. Lima, S. Bertini, R.R. de Lima, K.A. Furuta, C.H. Mapa, L. Roschel, E. Oliveira

**Affiliations:** 1 Saude iD, Grupo Fleury, São Paulo, SP, Brasil

**Keywords:** Covid-19, SARS-CoV-2, Disease severity, Telemedicine, Infectious diseases

## Abstract

In early 2021, Brazil saw a dramatic recurrence in Covid-19 cases associated to the spread of a novel variant of the SARS-CoV-2 virus, the P1 variant. In light of previous reports showing that this variant is more transmissible and more likely to infect people who had recovered from previous infection, a retrospective analysis was conducted to assess if the early 2021 Covid-19 wave in Brazil was associated with an increase in the number of individuals presenting with a more severe clinical course. Fifty-one thousand and fourteen individuals who underwent telemedicine consultations were divided into two groups: patients seen on or before January 31, 2021, and on or after February 1, 2021. These dates were chosen based on the spread of the P1 variant in Brazil. Referral to the emergency department (ED) was used as a marker of a more severe course of the disease. No differences were seen in the proportion of patients referred to the ED in each group nor in the odds ratio of being referred to the ED from the 1st of February 2021 (OR=0.909; 95%CI: 0.81-1.01). Considering the entire cohort, age had an impact on the odds of being referred to the ED, with individuals older than 59 years showing twice the risk of the remaining population and those less than 19 years showing a lower risk.

## Introduction

The Covid-19 pandemic has pushed health systems worldwide to their limits. In Brazil, the first case of severe acute respiratory syndrome coronavirus 2 (SARS-CoV-2) infection was reported on February 26, 2020, in the city of São Paulo (1). This was followed by an exponential increase in Covid-19 cases leading to the worst sanitary crisis in Brazil’s history.

Despite a reduction in the number of cases reported in the last trimester of 2020, Brazil was again hit by a dramatic recurrence of Covid-19 cases in early 2021. This wave of the pandemic is attributed to the spread of the P1 variant, which was initially identified in the state of Amazonas in December 2020, and systematically reported in other areas of the country starting in February 2021 (2).

Early reports show that the P1 variant is up to two times more transmissible and has higher infection rates among individuals previously infected with non-P1 variants (2,3). Despite anecdotal reports of an increase in the proportion of individuals with a more severe clinical course (4), it is still unknown whether the early 2021 Covid-19 wave in Brazil was associated with an increase in the number of severe cases.

The Saúde iD platform is part of the Fleury Group, one of Brazil’s oldest and largest private health companies, and has been offering telemedicine consultations to a population of 3.6 million people since June 2020. Telemedicine, which was subject to emergency regulation in Brazil after the Covid-19 pandemic, is now an important source of care for Covid-19 patients and for patients who are unable to leave home or feel unsafe to do so. This study sought to shed light on the question of whether the most recent wave of Covid-19 in Brazil, attributed to the P1 variant, was indeed associated with higher disease severity. The results of Covid-19 telemedicine consultations were used to assess whether the cases seen during the early 2021 wave were associated with an increase in emergency department (ED) referrals, used as an indicator of severity, as described in the next section.

## Material and Methods

All patients seen by Saúde iD telemedicine who received a diagnosis of Covid-19 during the teleconsultation were included in the analysis. To reduce selection bias, we have only included cases with a confirmed diagnosis of Covid-19 based on the International Classification of Diseases code U07.1 and on the confirmed diagnosis on our medical records.

Data on sex, age, and the outcome of the consultation were collated. Subsequently, two groups were created based on the date of the telemedicine appointment: one group included patients seen on or before January 31, 2021, and the other group patients seen on or after February 1, 2021. This date was chosen to reflect most accurately the new Covid-19 wave.

Three possible outcomes were recorded after all consultations: discharge, referral to a specialist, and referral to the ED. Although physicians are instructed to use their clinical judgment when deciding on the best outcome for each patient, Saúde iD provides an internal guideline to aid doctors in Covid-19 consultations, which is based on guidelines published by the Brazilian Society of Infectious Diseases (SBI) and the National Institutes of Health (5,6). According to the guidelines, mild cases should self-isolate for at least 10 days, take symptomatic treatment if needed, and be alert to warning signs that would require self-referral to the ED. Patients were referred to the ED if one or more of the following signs/symptoms were present: fever for more than 48 h, cyanosis or dyspnea or SpO_2_ <95%, tachypnea, persistent chest tightness, signs of hypotension (fainting sensation, cold and sticky skin), and/or signs of respiratory distress (broken speech, nose flaring, sternal retraction, use of accessory breathing muscles). The outcome “referral to specialist” was used if patients showed no warning signs and needed elective specialist care.

Statistical analysis was conducted using SPSS^®^ (subscription version, IBM Corporation, USA). Proportions were compared using the chi-squared test. Odds ratio and 95% confidence intervals were calculated for ED referral. Data for age were not normally distributed and therefore were compared using the Mann-Whitney U test. A P value of less than 0.05 was considered significant. All patients seen by Saúde iD telemedicine signed a consent form following the Brazilian General Law of Data Protection (Lei Geral de Proteção de Dados). No study-specific informed consent was required since data used in this study were routinely acquired medical data. The project was approved by the Fleury Group Ethics Committee.

## Results

**Figure 1:**
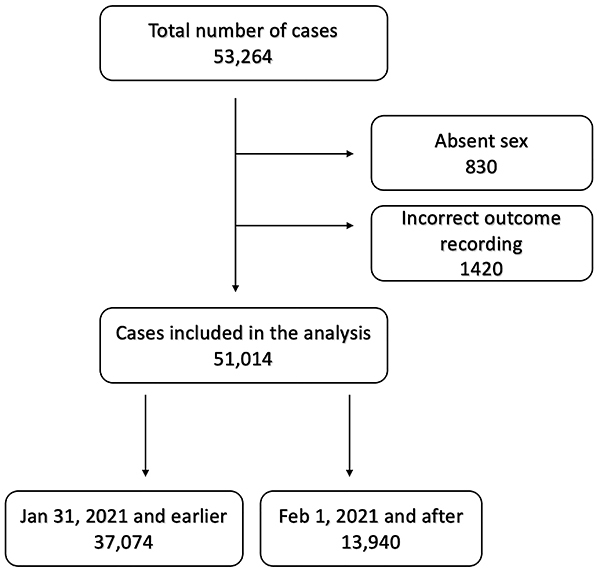
Flowchart of cases included in the analysis.

A total of 53,264 patients who received a diagnosis of Covid-19 (ICD U07.1) were identified. Eight-hundred and thirty cases were excluded because sex was not recorded, and 1420 cases had inappropriate recording of outcomes and were also excluded from the analysis. The remaining 51,014 cases were included in the final analysis and divided into two groups as described in the Methods section: 37,074 patients were seen on or before January 31, 2021 and 13,940 were seen on or after February 1, 2021 (Figure 1).

The majority of patients were women (55.6% of the total population; 28,343 individuals) and the mean age of the study population was 34.3 years (median 34; range 0-97 years) (Table 1).

**Table 1:** Demographic data of the entire cohort assessed by telemedicine at different times for Covid-19.

	Total	January 31st and earlier	February 1st and after	P value
N	51,014	37,074	13,940	
Female sex (%)	55.6	55.8	55	0.105*
Age in years				0.233**
Mean	34.39	34.50	34.03	
Median	34.0	34.0	34.0	
Range	0-97	0-97	0-92	

*Chi-squared test.

**Mann-Whitney U test.

**Figure 2:**
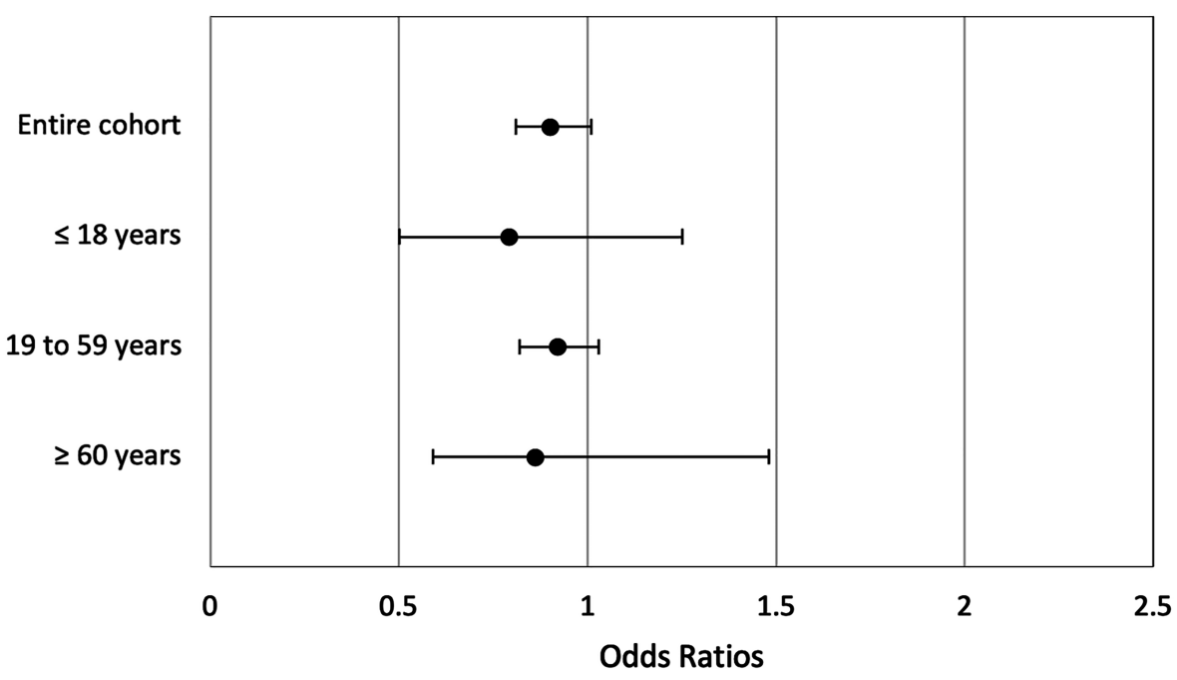
Odds ratios and 95% confidence intervals of emergency department referral for patients seen on or after February 1, 2021. For more details, see Supplementary Table S1.

No differences were seen in consultation outcomes: a similar proportion of patients was discharged, referred to a specialist, or referred to the ED in both groups (Table 2). The most common outcome was discharge (96.1% before February 1st and 96.4% after), followed by referral to ED (3.6 and 3.3%) and referral to specialist (0.2% for both groups). The odds ratio of ED referral after February 1st was also calculated for both groups, and again no differences were seen, showing a similar risk of ED referral in the early 2021 wave compared to previous waves (Figure 2).

**Table 2:** Comparison of the outcome between groups assessed by telemedicine at different times for Covid-19 in Brazil.

	January 31st and earlier	February 1st and after	P value
Entire cohort			
Discharge	35,638 (96.1%)	13,445 (96.4%)	0.217
Referral to specialist	83 (0.2%)	31 (0.2%)	
Referral to emergency department	1353 (3.6%)	464 (3.3%)	
≤18 years			
Discharge	1971 (96.2%)	1203 (96.9%)	0.594
Referral to specialist	19 (0.9%)	10 (0.8%)	
Referral to emergency department	58 (2.8%)	28 (2.3%)	
19-59 years			
Discharge	32,989 (96.2%)	11,964 (96.5%)	0.414
Referral to specialist	57 (0.2%)	20 (0.2%)	
Referral to emergency department	1241 (3.6%)	417 (3.4%)	
≥60 years			
Discharge	678 (91.7%)	278 (93.3%)	0.519
Referral to specialist	7 (0.9%)	1 (0.3%)	
Referral to emergency department	54 (7.3%)	19 (6.4%)	

Data are reported as number and percentage. The chi-squared test was used for statistical analyses.

Considering the higher risk of hospitalization and a more severe clinical course in older individuals, the entire cohort was divided into three age groups for further statistical analysis: 18 years old and younger (n=3289; mean age 10.4 years; 51.2% female); 19 to 59 years old (n=46,688; mean age 35.3 years; 56% female); and 60 years and older (n=1037; mean age 65.6 years; 50.1% female). Details on the demographics of each age group are shown in Supplementary Table S1. Similar to the previous analysis, the comparison between groups showed no differences in the proportion of each outcome nor in the odds ratio of ED referral before or after February 1st (Table 2, Figure 2, and Supplementary Table S2).

**Figure 3:**
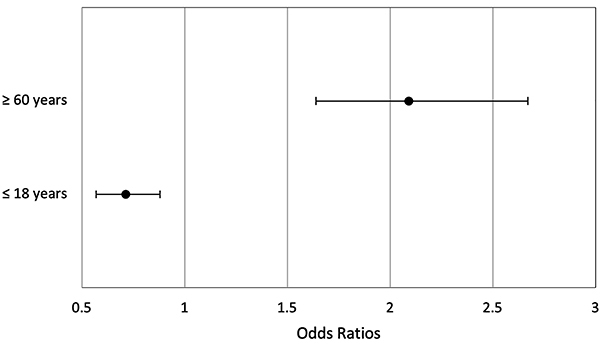
Odds ratios and 95% confidence intervals of emergency department referral for individuals in the older and younger age groups for the entire duration of the study.

Individuals in the older age group (≥60 years) were twice more likely to be referred to the ED compared to the two other groups (OR=2.09; 95%CI: 1.64-2.67; χ^2^: P<0.001). On the other hand, individuals in the younger age group (≤18 years) had a lower risk of being referred to the ED (OR=0.713; 95%CI: 0.57-0.88; χ^2^: P=0.003) (Figure 3). Within-group comparisons of the outcome and odds ratio of ED referral based on date of appointment (≤31st of January and ≥1st of February) were also conducted and no differences were found.

## Discussion

This retrospective analysis of patients receiving care through telemedicine did not show that the early 2021 Covid-19 wave in Brazil was associated with an increase in the number of more severe cases, as measured by ED referral. The analysis of the entire population, regardless of date of consultation, showed that older individuals were at a higher risk of presenting with a more severe clinical course, as reported by previous studies (7,8).

Younger patients tend to be more willing to adopt new technologies and are therefore more likely to be overrepresented in telemedicine consultations and contribute to selection bias. However, the mean age of the study cohort is similar to the national average, according to the Brazilian Institute of Geography and Statistics (IBGE). Furthermore, although there is a higher proportion of women in the Brazilian population, it was even higher among study participants (51.8% in Brazil compared to 55.6% of participants). Inappropriate recording of sex and outcomes led to the exclusion of 2,250 cases. Since this number corresponds to 4.2% of the original cohort, it is unlikely that the exclusion resulted in bias.

To the best of our knowledge, this is the first study to compare the proportion of ED referrals after teleconsultations as an indicator of severity. Clinical data on disease severity is of paramount importance, particularly after previous publications reported higher transmissibility and higher infection rates in individuals with previous infections by other SARS-CoV-2 variants (2). In this study, a higher severity of Covid-19 was not detected and referral to ED rates were similar in both groups, leading to the conclusion that the most recent wave of the disease was not associated with a more severe clinical course. Similar results were also seen for each of the three age groups analyzed, corroborating the main finding. Since this is the first study to report such finding, the results need to be confirmed by other studies.

The early 2021 spread of Covid-19 in Brazil could be attributed to diverse factors, such as the relaxation of measures to control dissemination of the virus throughout the country (e.g., social distancing, relaxation of mask use, and resumption of mass gatherings), but the most important factor appears to be the emergence of the P1 variant in the state of Amazonas (2). A previous serological study using blood samples from Manaus, the Amazonas state capital, showed that nearly 75% of the population had been infected by the SARS-CoV-2 by October 2020 (9). The P1 variant, which was absent in samples collected in Manaus from March to November 2020, was identified for the first time in the city in December 2020. This was followed by a dramatic increase in case reports resulting in the collapse of the city's health care system. In parallel, the prevalence of the P1 variant increased sharply, accounting for 52.2% of the samples collected in December 2020 and 85.4% of the samples from January 2021 (2,10,11).

The P1 variant spread fast to other areas of the country, reaching the Southeast region in January as shown by reports of local transmission (12). By March, the P1 was already the most prevalent variant in Sao Paulo. Genotypic analysis revealed an increase in the prevalence of the P1 variant from 78.6% in the first week of March to 91.7% in the second week (13). The proliferation of the P1 variant appears to be the consequence of 17 new mutations, some of which increased the capacity of its spike protein to bind to the ACE-2 receptor and infect host cells. The variant not only appears to be approximately two times (1.7 to 2.4) more contagious than previous variants, but it can also evade the host’s immunological response more effectively. Previous non-P1 infections provide an immunological response against the P1 variant that is only 54-79% as effective as the response against non-P1 variants (2).

Due to the absence of genotypic data to identify SARS-CoV-2 variants in this study, a date had to be chosen to separate the presumed non-P1 waves from the early 2021 wave (when most cases were associated with the P1 variant). Considering the Ministry of Health’s daily reports of Covid-19 cases and deaths and studies that have used genotyping to identify the spread of the different variants in the country (2,11,13), it was assumed that consultations that occurred on or after February 1 were more likely to be cases of infection with the P1 variant. However, it is possible that a significant number of non-P1 variant cases were included in this group, making the selection method an important caveat of this study. Nonetheless, considering the impact of the early 2021 wave of Covid-19 on the Brazilian health system, any clinical data, particularly on case severity, is welcome.

Another limitation is the use of the outcome “ED referral” as an indicator of severity. To increase the objectivity of this variable, doctors are advised to follow Saúde iD's internal Covid-19 guideline, which is based on national and international protocols and clearly states symptoms and signs associated with a more severe clinical course. The guideline also states the reasons for immediate referral to a clinical assessment in the ED. Although it cannot be guaranteed that all physicians adhered to the guideline, its availability has likely reduced the misidentification of severe cases, or conversely, the failure to refer a case requiring hospitalization to the ED. Another issue that could have influenced the outcome is the absence of on-demand laboratory tests that could identify severe cases that were missed by the physician's assessment due to the paucity of clinical signs/symptoms.

Selection bias could have affected the results in other ways. Patients with more severe forms of the disease would be more likely to bypass telemedicine and self-refer to the ED. The majority of the study population was in the 19-59 years of age group. The small number of patients in the other groups (≤18 years and ≥60 years) limits the generalizability of our findings to other age groups. Another important issue is that Brazil is a continental country with large income disparities, which contributes to different rates of adoption of new technologies, such as telemedicine. Saúde iD is a private company that provides telemedicine services to health insurance customers, hence our conclusions cannot be extrapolated to other demographic groups, such as the users of the public National Health System (SUS). Only 28.5% of Brazilians have health insurance, further limiting the generalization of our findings to the entire Brazilian population (14).

Reliable findings of this study are the higher risk of older patients of being referred to the ED and the lower risk for individuals with less than 19 years of age, which is in line with previous findings (15) and therefore support the reliability of the outcome “referral to ED” as an indicator of severity.

Future studies should follow patients longitudinally and assess in-patient morbidity and mortality rates to provide a definitive answer to this question. The findings reported here highlight the importance of telemedicine in providing quality medical care during the Covid-19 pandemic.

### Conclusions

This retrospective analysis of individuals with Covid-19 receiving care through private telemedicine did not show an increased risk of developing a more severe course of the disease in the early 2021 wave in Brazil. In both periods of assessment, children and teenagers were more likely to present with a more benign course and older individuals were at higher risk of severe disease, confirming previous findings.

## Supplementary Material

Click here to view [pdf].
